# A case with hepatic portal vein gas who required delayed elective surgery

**DOI:** 10.1016/j.ijscr.2019.10.085

**Published:** 2019-11-06

**Authors:** Kou Ikegame, Yuji Iimuro, Kazushige Furuya, Hiroshi Nakagomi, Masao Omata

**Affiliations:** a Department of Surgery, Yamanashi Prefectural Central Hospital, Japan; b Internal Medicine, Yamanashi Prefectural Central Hospital, Japan

**Keywords:** HPVG, hepatic portal vein gas, CT, computed tomography, APACHE II, Acute Physiology and Chronic Health Evaluation II, Case report, Hepatic portal vein gas, Delayed elective surgery, Acute Physiology and Chronic Health Evaluation (APACHE) II score

## Abstract

**Introduction:**

Hepatic portal venous gas (HPVG) is believed to be an indication for emergent surgery because it is associated with high mortality rate. However, the recent increase in the use of modern abdominal computed tomography (CT) has resulted in the detection of HPVG in more benign conditions. Therefore, the decision-making process whether we chose emergent surgery or conservative treatment without surgery is important for the patients with HPVG.

**Case presentation:**

An 84-year-old male was referred to our hospital due to the sudden onset of abdominal pain and massive hepatic portal vein gas on emergent CT. The Acute Physiology and Chronic Health Evaluation (APACHE) II Score was calculated as 17; slightly elevated comparing with the other cases who were successfully treated without surgery. Although the PHVG was remained at follow up CT on the next day after the onset, the symptoms were improved. We selected conservative treatment without emergent surgery and he discharged on 9^th^ day after the onset. However, he was suffered from right lower abdominal pain and vomiting and admitted our hospital on 23th day. He developed ischemic intestinal stenosis and underwent a surgery of partial resection of ileum.

**Conclusions:**

The clinical finding of this case showing subtle differences from cases who were successfully treated without surgery. We hope this report will help physician’s decision-making process for HPVG.

## Highlights

• Hepatic portal venous gas (HPVG) is believed to be an indication for emergent surgery because it is associated with high mortality rate.
• The recent increase in the use of modern abdominal CT has resulted in the detection of HPVG in more benign conditions.
• The decision-making process whether we choose emergent surgery or conservative treatment without surgery is important for the patients with HPVG.
• The case with portal hepatic vein gas (HPVG) presented in this article required delayed elective surgery after conservative treatment.
• This case show the subtle difference comparing with the other case who were successfully treated without surgery.

## 1 Introduction

The presence of hepatic portal venous gas (HPVG) had previously been considered to reflect abdominal severe events, such as intestinal necrosis that required emergent surgery and associated with a high mortality rate [1]. However, the recent increase in the use of modern abdominal CT has resulted in the detection of HPVG in more benign conditions [2]. As a result, there are many reports of patients with HPVG who recovered after conservative treatment [3]. Indeed, in 1978, a review of early studies of HPVG found that HPVG was associated with a mortality rate of 75%, while a survey in 2001 revealed that the overall mortality rate was 39% [4].

The decision-making process whether to choose emergent surgery or conservative treatment without surgery is important for the patients with HPVG. The case presented in this article required delayed elective surgery after conservative treatment show subtle differences in clinical finding and laboratory data from cases who were successfully treated without surgery or cases required emergent surgery. We would like to compare the clinical data of the other 17 cases in our hospital to focus on the subtle differences of clinical finding of this case.

This work has been reported in line with the SCARE criteria [5].

## 2 Case presentation

**Fig. 1:**
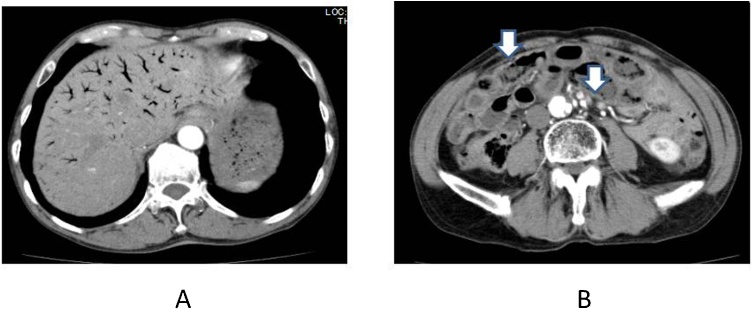
Contrast-enhanced CT on admission revealed hepatic portal vein gas in whole liver (1A) and intestinal pneumatosis at ileum (1B). White arrows indicate pneumatosis of intestine.

**Fig. 2:**
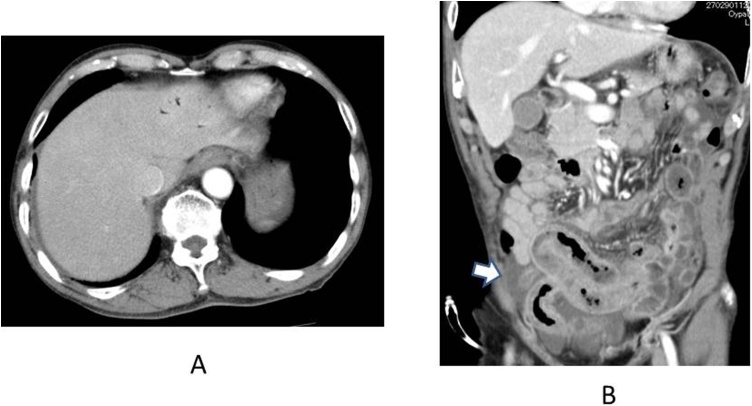
CT on the next day after the onset. Small HPVG remained at lateral segment (2A) and increased ascites (2B: indicated by white arrow). Intestinal pneumatosis was distinguished.

An 84-year-old male had visited to a hospital due to the sudden onset of abdominal pain. He had developed massive hepatic portal vein gas on emergent CT and referred to our hospital. He had been treated for the cerebral infarction and atrial fibrillation, and had history of inguinal hernia repair and appendectomy. Physical finding at administration showed slight distension and tenderness of lower abdomen but no tenderness. Vital signs were stable with 130/84 mmHg of blood pressure, 71/min of pulse rate and 36.9 °C of body temperature. Laboratory data showed as follows; white blood cell (WBC) 13,300/μl, c-reactive protein (CRP) 37 mg/dl, base excess (BE) 3, total bilirubin

(T-Bil) 0.0 mg/dl, creatine kinase (CK) 53 mg/dl, aspartate aminotransferase (AST) 16 U/l, alanine aminotransferase (ALT) 123 U/ｌ, lactate dehydrogenase (LDH) 474U/l, creatinine (Cr) 1.0 mg/dl. The Acute Physiology and Chronic Health Evaluation II (APACHE II) score was calculated as 17. Enhanced CT on admission revealed hepatic portal vein gas in whole liver and intestinal pneumatosis at ileum (Fig. 1A and B). We decided to make conservative therapy with heparinization without emergent surgery. The CT on the next day showed small HPVG remained at lateral segment and increased ascites. Intestinal pneumatosis was distinguished and symptoms were improved. Feeding was started on 5^th^ day after onset and he was discharged on 9^th^ day. However, he was suffered from right lower abdominal pain and vomiting and admitted our hospital on 23th day (Fig. 2).

**Fig. 3:**
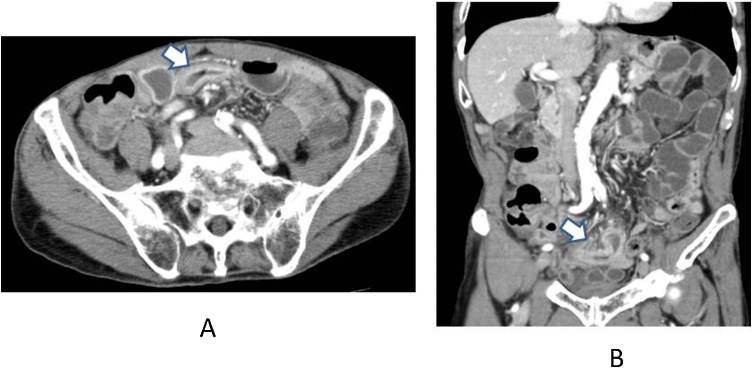
CT on 23th day after onset of HPVG. CT revealed thickness of intestinal wall which was a same portion of intestinal pneumatosis and fluid collection in oral intestine (Fig. 3A and 3B: indicated by white arrow).

**Fig. 4:**
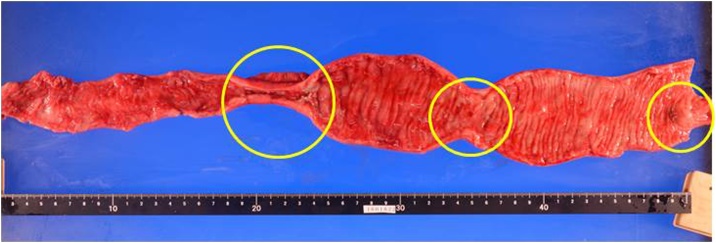
Surgical Specimen. The resected ileum was 40 cm of length and had segmental stenosis at three portions (yellow circles).

CT revealed thickness of intestinal wall which was a same portion of intestinal pneumatosis and fluid collection in oral intestine (Fig. 3A and B). We decided to perform laparotomy under the diagnosis with bowel obstruction and made partial resection of ileum. The resected ileum was 40 cm of length and had segmental stenosis at three portions (Fig. 4). Pathologic diagnosis was an ischemic intestinal stenosis. Clinical course after the operation was no eventful.

## 3 Discussion

The exact etiology of hepatic portal venous gas (HPVG) is still unclear, because only a small number of case reports and systematic reviews are available [1,2,6]. HPVG was first reported by Wolfe and Evance in 1955 [7]. In general, HPVG is caused by intestinal ischemia, but several reports have discussed the development of HPVG in patients with other non-ischemic conditions including ulcerative colitis [8], Crohn’s disease [9], enteritis [10] cholangitis [11,12], neobladder obstruction [13] and gastric ulcer [4].

We treated 18 cases with HPVG from January, 2014 to January, 2017, which were divided into four groups according to the severity of HPVG: alive without surgery, alive after elective surgery, alive after emergent surgery; and dead with/without emergent surgery (Table 1). Emergent surgery was required for 6 cases (30%) and two cases died 33 and 46 operative days. While 11 cases (61%) were recovered without surgery. Obvious difference of APCHE II score was seen between cases required emergent surgery and recovered without surgery (23 ± 5 vs 15, p < 0.05). It is noted that the APACHE II score of this case was 17, valued at intermediate between the values of both groups.

**Table 1:** Cases developed hepatic portal vein gas (HPVG) during 2014–2017.

No	Sex	Age	Causative Disease	Surgery	Surgical Procedure	Prognosis (days of death)	APACHE II
1	M	80	massive intestinal necrosis	emergent	massive intestinal necrosis	death (33)	25
2	M	84	SMA occlusion	emergent	resection of massive intestine and right side colon	death (46)	33
3	M	63	intestinal perforation	emergent	resection of small intestine 20 cm	alive	19
4	F	80	intestinal perforation due to malignant lymphoma	emergent	rghi side colectomy	alive	23
5	M	77	ischemic intestine	emergent	resection of small intestine 15 cm	alive	20
6	F	72	ischemic intestine	emergent	adhesiolysis	alive	20
7	M	85	ischemic intestine → stenosis	elective^a^	resection of small intestine 40 cm	alive	17
8	M	73	unknown	none	na	alive	15
9	M	68	gastric erosion	none	na	alive	15
10	F	86	SMA dissection	none	na	alive	15
11	F	88	unknown	none	na	alive	15
12	M	77	ischemic intestine	none	na	alive	15
13	M	68	unknown	none	na	alive	15
14	F	60	ischemic intestine	none	na	alive	15
15	F	55	ischemic intestine	none	na	alive	15
16	F	82	small bowel obstruction	none	na	alive	15
17	M	69	small bowel obstruction	none	na	alive	15
18	M	71	ischemic intestine	none	na	alive	15

na; not applicable.

a Presented case.

Indeed, the utility of APACHE II score for determining the treatment for HPVG [14], and correlation with the prognosis of patients who require intensive care [15,16] were reported. Thus, this score, which assesses the whole body condition, could be useful for selecting surgery or conservative treatment [14,17,18].

Most of our cases were associated with intestinal ischemia, which was indicated by the CT finding of intestinal ischemia and mesenteric vein gas or intestinal pneumatosis. If we missed these findings, a correct diagnosis would have been difficult to make.

The role of CT in predicting the prognosis of HPVG has been reported, in a study that emphasize the importance of the extent of hepatic portal gas and the existence of intestinal pneumatosis were emphasized [19]. In addition, we would like to recommend follow-up CT after a few days seems to be most useful for the management for HPVG. We could find a change of CT finding for 6 cases including presented case (Table 2). Presented case is only one case whose follow up CT showed remained portal hepatic gas, in contrast with other cases whose portal hepatic gas was distinguished at CT on 1–3 days after the onset. When the physical findings associated with the need for emergent surgery are not observed, HPVG or mesenteric vein gas quickly disappeared in a few days.

**Table 2:** The change of CT finding of HPVG cases without surgery (n = 6).

	day 0	day 1–3
intestinal ischemia	2	0
hepaticportal vein gas	6	1^a^
intestinal pneumatisis	6	2
mesenteric vein gas	3	0
ascites	2	5^a^

a hepatic portal vein gas was remained and ascites increased at follow up CT in presented case.

In summary, presented case was an only one who required elective surgery among 18 cases with HPVG in our experience. This case had subtle differences of clinical findings apart from the other cases. The APACHE II Score was slightly elevated and the PHVG was remained at follow up CT on the next day after onset comparing with the other case who were successfully treated without surgery.

We hope the clinical finding of this case required delayed elective surgery will help the physician’s decision-making process for HPVG.

## 4 Conclusions

The case presented in this article required delayed elective surgery after conservative treatment show the subtle difference comparing with the other case who were successfully treated without surgery. The APACHE II Score was slightly elevated and the PHVG was remained at follow up CT on the next day after the onset. We hope this report will help the physician’s decision-making process for HPVG.

## Sources of funding

We did not receive any specific grant from funding agencies in the public, commercial, or not-for-profit sectors.

## Ethical approval

The case report was approved by the institutional review board at Yamanashi Prefectural Central Hospital.

## Consent

Written Informed Consent was obtained from the patient for publication of this case and any accompanying images. A copy of the written consent is available for review by the Editorial-in-Chief of this journal.

## Author contribution

KI, TI, KF, HN, YO, and MO conceived of this case presentation and drafted the manuscript. AT, HW, TN, HN, KM, HM, MY, MI and HM participated in the treatment of this case. All authors read and approved the final manuscript.

## Registration of research studies

This is mandatory for human studies only.　

We obtained written informed consent by the patients, concerning this publication.

We attached the copy of the written informed consent.

I obtained UIN “researchregistry5139”.

## Guarantor

Hiroshi Nakagomi and Masao Omata have accept full responsibility for this work and controlled the decision to publish.

## Provenance and peer review

Not commissioned, externally peer-reviewed.

## Declaration of Competing Interest

The authors declare no conflicts of interest.
